# Materials utilizing supramolecular host-guest binding for gold extraction†

**DOI:** 10.1039/d5ra02404h

**Published:** 2025-04-22

**Authors:** Michaela Šusterová, Vladimír Šindelář

**Affiliations:** a Department of Chemistry, Faculty of Science, Masaryk University Kamenice 5 625 00 Brno Czech Republic; b RECETOX, Faculty of Science, Masaryk University Kamenice 5 625 00 Brno Czech Republic sindelar@chemi.muni.cz

## Abstract

The dicyanoaurate anion plays a central role in the gold mining industry. Its recovery from an aqueous solution is dominantly achieved using activated carbon, which, however, suffers from several drawbacks. Herein, we report a simple preparation of a new material containing the bambusuril macrocycle physically sorbed on the surface of silica gel particles. We utilized the ability of bambusuril to form a unique material that extracts dicyanoaurate from an aqueous solution *via* supramolecular host-guest interactions. Notably, the material selectively removed dicyanoaurate over dicyanoargentate. Equilibrium sorption and desorption of the anion were achieved within minutes at ambient temperature, significantly outperforming activated carbon.

## Abstract 2

Molecules of the bambusuril macrocycle attached to silica gel particles effectively removed dicyanoaurate anions from water through host-guest supramolecular interactions.

## Graphical abstract

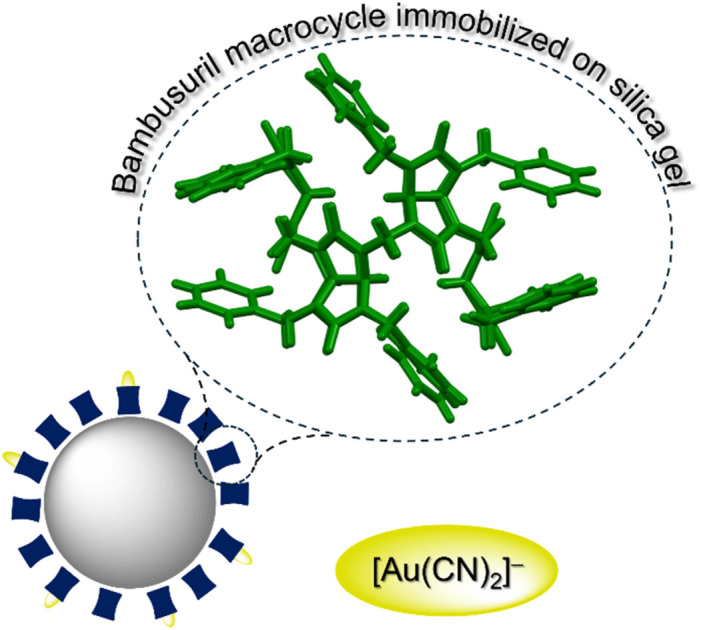

## Introduction

Gold is an indispensable and precious metal that has found application in many fields, including jewelry, electronics,^1^ chemical synthesis,^2–4^ catalysis,^5,6^ nanotechnology,^7^ and medicine.^8^ Supramolecular host-guest approaches for gold mining and recycling as an alternative to traditional technologies are currently of high interest. Successful approaches dealing mainly with supramolecular interactions of Au(iii) anions, such as AuBr_4_^−^, have used cucurbiturils,^9,10^ cyclodextrins,^11^ and other molecules^12^ to selectively precipitate Au(iii) assemblies. However, industrial gold production is mainly based on the cyanide process, involving the treatment of ores with a cyanide solution resulting in the formation of dicyanoaurate(I), [Au(CN)_2_]^–^.^13^ The anion is removed from the solution by its sorption onto activated carbon following anion stripping from the material. The stripping of [Au(CN)_2_]^–^ requires elevated temperature and pressure, which increases the cost of gold production.^13,14^ Recently, Stoddart and co-workers and later our group recognized α-cyclodextrin^15^ and bambusuril macrocycles,^16^ respectively, as suitable hosts for [Au(CN)_2_]^–^ and demonstrated that the selective stripping of the anion from activated carbon can be achieved using these macrocycles at room temperature.

The use of activated carbon is not only associated with expensive stripping but is further complicated by (i) long sorption and stripping time of [Au(CN)_2_]^–^, (ii) low selectivity for [Au(CN)_2_]^–^ over [Ag(CN)_2_]^–^, and (iii) necessity to reactivate carbon in a kiln requiring high energy consumption.^13,17^ In addition to activated carbon, the gold mining industry currently uses ion-exchange resins.^18,19^ There are several such resins available in the market, and some plants have already implemented these adsorbents in their processes. Nevertheless, both substrates face issues related to stripping and subsequent recycling of materials, involving elevated temperatures and treatment with toxic solvents.

**Fig. 1:**
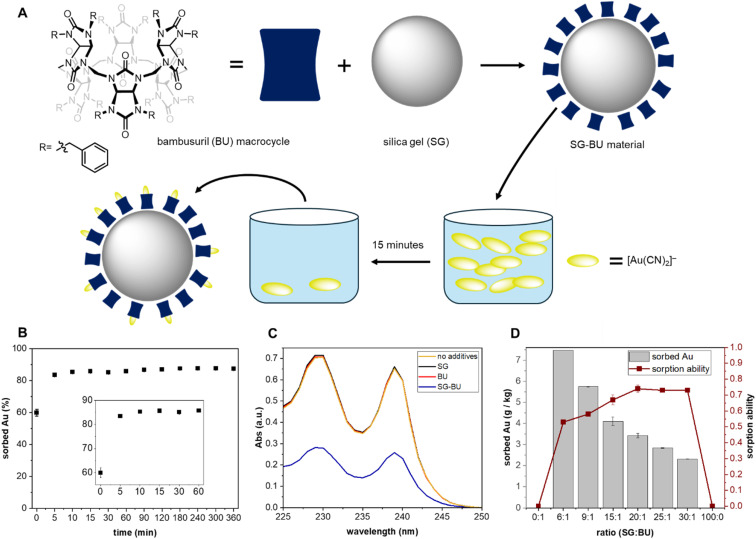
(A) Preparation of the SG-BU material, and illustration of the sorption experiment. (B) Sorption kinetics expressed as time dependence of the sorption efficiency (the weight fraction of gold removed from the solution by the material with the silica gel/BU ratio of 9 : 1 *w*/*w*). (C) UV-vis spectra of water solution of K[Au(CN)_2_] (1 mM) in the absence (no additives) and in the presence of silica gel (SG), BU, and the SG-BU material. (D) Dependence of binding potency (sorbed Au) and sorption ability (molar ratio of sorbed anions and bambusurils on the material) on the silica gel/BU w/w ratio.

Bambusuril macrocycles and [Au(CN)_2_]^–^ form inclusion complexes with micromolar stability in water, and the macrocycle selectivity for [Au(CN)_2_]^–^ over [Ag(CN)_2_]^–^ is two orders of magnitude.^16^ Therefore, we envisioned bambusuril-based materials for the effective uptake of [Au(CN)_2_]^–^ from aqueous media and their potential as an activated carbon substitute in gold mining. Herein, we report a straightforward preparation of silica gel materials with physically attached hydrophobic dodecabenzylbambusuril (BU, Fig. 1A).^20^ This material exhibited highly efficient and selective sorption of [Au(CN)_2_]^–^ from water. Fast elution of [Au(CN)_2_]^–^ from the material and simultaneous recycling of the material using a NaCl solution were also demonstrated.

## Results and discussion

The material for [Au(CN)_2_]^–^ sorption was prepared simply by mixing commercially available silica gel with BU in chloroform (Fig. 1A). Evaporation of the solvent produced a material containing bambusuril deposited on the surface of silica gel particles. BU is highly hydrophobic, and it is essentially insoluble in water. Therefore, pure bambusuril remained floating as a white powder on the water surface even after turbulent mixing. However, when combined with silica gel, the properties of the resulting materials change according to the silica gel/BU ratio. While the material with a high BU content still showed the separation of the macrocycle on the water surface, the materials with the silica gel/BU weight ratio exceeding 6 : 1 appeared as compact sediments on the bottom of the vial filled with water. The water insolubility of BU alone and in the presence of silica gel was confirmed by total organic carbon analysis (Table S1†). The prepared materials remained stable up to 320 °C without significant decomposition, as measured by thermogravimetric analysis (Fig. S2†).

We tested the prepared materials for the uptake of [Au(CN)_2_]^–^ from water. For all experiments, we used an aqueous solution of [Au(CN)_2_]^–^ (1.0 mM, 3.0 mL, 3.0 μmol), and the actual concentration of [Au(CN)_2_]^–^ was monitored by UV-vis or inductively coupled plasma (ICP) spectroscopy. First, we investigated the time necessary to reach the equilibrium sorption of the materials. The material with the 9 : 1 silica gel/BU weight ratio (SG-BU (9 : 1), 100 mg, 5.0 μmol of BU) was mixed with the [Au(CN)_2_]^–^ aqueous solution, and the decrease in the anion concentration was monitored over time. It was found that 15 minutes is sufficient to reach an equilibrium concentration of the anion in the solution (Fig. 1B). This time was then used in all subsequent sorption experiments. The time required for the sorption of the anion by activated carbon is usually several hours to days, and equilibrium loading is reached only after weeks or even months.^21^ Our experiment showed that the equilibrium sorption of the anion by activated carbon was not achieved within 3 hours of the experiment (Fig. S8†).

Next, we investigated the influence of the material composition on its ability to capture [Au(CN)_2_]^–^ from its water solution. The addition of either the BU macrocycle or silica gel to the water solution of [Au(CN)_2_]^–^ did not result in any decrease in the two characteristic absorption bands of the anion (229 and 239 nm) in the UV-vis spectra (Fig. 1C), indicating that these materials are unable to extract [Au(CN)_2_]^–^ from its water solution. This is in agreement with the hydrophobic nature of BU, for which the macrocycle is insoluble and thus inaccessible to the anion, and the inability of silica gel to bind the anion. In contrast, the presence of all materials with a silica gel/BU weight ratio greater than 6 : 1 resulted in a significant intensity decrease in the [Au(CN)_2_]^–^ absorption bands, indicating the sorption of the anion by the materials. To compare the sorption characteristics of these materials, we used them in such quantities that they all contained the same amount of BU (5.0 mg, 2.5 μmol) and added them to the [Au(CN)_2_]^–^ solution (1.0 mM, 3.0 mL, 3.0 μmol). First, we calculated how many anions per molecule of bambusuril is bound to the material (sorption ability). We observed that the material sorption ability increased from 53% to 74% when the silica gel/BU ratio increased from 6/1 to 20/1 and did not significantly differ for materials with lower BU content, that is SG-BU (20 : 1), (25 : 1), and (30 : 1) (Fig. 1D). This is probably caused by a better distribution of bambusuril molecules on the silica gel surface when more silica gel particles are available. The amount of Au sorbed per amount of used material decreased from 7.47 to 3.43 g kg^−1^ when the silica gel/BU ratio increased from 6 : 1 to 20 : 1 (Fig. 1D). This was expected because the materials used for the sorption contained the same amount of BU, and only the amount of inactive silica gel in the material varied.

**Fig. 2:**
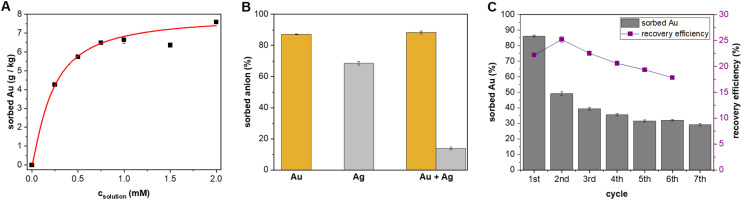
(A) Langmuir-type isotherm of sorption. (B) Weight fraction of gold/silver removed from 1 mM water solution of K[Au(CN)_2_], K[Ag(CN)_2_], and the mixture of both anions by the SG-BU (9 : 1) material (100 mg). (C) Sorption efficiency (weight fraction of gold removed from 1 mM water solution of K[Au(CN)_2_]) and recovery efficiency (weight fraction of gold removed from the SG-BU (9 : 1) material after treatment with NaCl solution) during several recycling steps.

The material with a silica gel/BU ratio of 9 : 1 (SG-BU (9 : 1)) was selected for subsequent measurements to compromise the sorption ability and the potency to bind Au. SG-BU (9 : 1) proved to be extremely powerful for the extraction of the anion. Adding excess material to the [Au(CN)_2_]^–^ aqueous solution decreased the gold concentration from 1 mM (180 mg L^−1^) to less than 0.1 μM (0.03 mg L^−1^) (Fig. S5†). To evaluate the sorption behavior of SG-BU (9 : 1), we conducted a sorption isotherm experiment in which a constant amount (10 mg) of the SG-BU (9 : 1) material was placed in solutions of different gold concentrations, and the amount of sorbed gold was monitored by UV-vis spectroscopy (Fig. 2A). The experimental data were fitted to the Langmuir model, yielding a material sorption capacity for Au of 7.9 ± 0.7 g kg^−1^ (Fig. S6†). The sorption capacity of activated carbon was found to be more than four times higher (Fig. S8†). We also calculated an association constant value of 5.9 × 10^3^ M^−1^ between [Au(CN)_2_]^–^ and BU attached to the material. This value is significantly lower compared to the one determined in water solution for different BU derivatives.^16^ The insolubility of bambusuril with its attachment on the material surface probably contributed to the observed lower binding. When a 0.1 mM [Au(CN)_2_]^–^ aqueous solution (3 mL) was used instead of the original 1 mM solution, 60% of the gold was removed by SG-BU (9 : 1) (10 mg) after 15 minutes (Fig. S9†). The quantitative uptake of gold was observed when SG-BU (9 : 1) (100 mg) was added (Fig. S10†). Notably, under the same conditions, only 10 mg of activated carbon removed more than 99% of the anion from the solution (Fig. S9†).

The main contaminant in the gold production is [Ag(CN)_2_]^–^, which is formed next to [Au(CN)_2_]^–^ during the cyanidation process.^22^ Silver complexes are adsorbed on activated carbon and must be eluted and recovered concurrently, which increases the cost of gold production.^17^ Therefore, we evaluated the material towards selective [Au(CN)_2_]^–^ uptake. Aqueous solutions of [Au(CN)_2_]^–^ (1 mM, 3 μmol) and [Ag(CN)_2_]^–^ (1 mM, 3 μmol) as well as an equimolar mixture of both anions (1 mM of each anion, 6.0 μmol) were shaken with SG-BU (9 : 1) (100 mg, 5.0 μmol of BU) for 15 minutes. ICP analysis revealed similarly high [Au(CN)_2_]^–^ uptake of 87% and 88% by the material immersed into the [Au(CN)_2_]^–^ solution in the absence and presence of [Ag(CN)_2_]^–^ (Fig. 2B). In contrast, the material removed 68% and 14% of [Ag(CN)_2_]^–^ from the solution in the absence and presence of [Au(CN)_2_]^–^, respectively. The superior preferential uptake of [Au(CN)_2_]^–^ over [Ag(CN)_2_]^–^ by the material is in agreement with the bambusuril selectivity of 100 towards the former anion.^16^

We also studied the recovery of the sorbed [Au(CN)_2_]^–^ from the material (Fig. 2C). After the sorption of [Au(CN)_2_]^–^, SG-BU (9 : 1) was washed with excess aqueous NaCl solution (3.5 wt%) at 23 °C, and the amount of [Au(CN)_2_]^–^ released from SG-BU into the solution was monitored as a function of time (Fig. S7†). Only 15 minutes were necessary to achieve a maximum recovery efficiency of 22% because no additional release of anion was monitored within the next 22 hours. The observed time for anion stripping is in contrast to several hours and harsh conditions used for the striping of anions from activated carbon.^17,23^ After treatment with NaCl solution, the material was reused for other 5 consecutive sorption cycles. The sorption efficiency of SG-BU (9 : 1) decreased from 86% to 49% after the first cycle and equilibrated at 29% after the 6th cycle (Fig. 2C). The recovery efficiency of the material decreased from 22% to 18% after the 6th cycle.

The stripping of [Au(CN)_2_]^–^ from the bambusuril-based materials and the simultaneous recycling of the material by NaCl are enabled by the interaction of the salt with the bambusuril. It has been reported that, in solution, bambusurils bind [Au(CN)_2_]^–^ as well as chloride inside their cavities, forming host-guest complexes with 1 : 1 binding stoichiometry. However, the bambusuril complex with [Au(CN)_2_]^–^ is three orders of magnitude more stable than the complex with chloride. Thus, stripping of [Au(CN)_2_]^–^ is achieved by washing the material with excess chloride, which exchanges for [Au(CN)_2_]^–^ in the bambusuril molecules attached to the silica surface. The material in the first sorption cycle contained bambusuril molecules without a bound anion. This is in contrast to all subsequent cycles in which the bambusuril molecules are occupied by either remaining [Au(CN)_2_]^–^ or chloride anions. Thus, the sorption capacity of SG-BU at the first cycle was significantly higher compared to other cycles. Despite this decrease, the sorption efficiency (49%) and recovery efficiency (18%) were relatively high considering the mild conditions and extremely short processing times.

## Conclusions

In conclusion, we report an SG-BU material for the recovery of [Au(CN)_2_]^–^, a process relevant to gold mining. The SG-BU preparation is very simple because it is based on the physical sorption of the BU macrocycle on silica gel. The material is stable in water because BU is insoluble in this medium and remains attached to silica gel. The sorption capacity of 7.9 g of Au per kg of the material containing 10 wt% BU was approximately four times lower than the capacity of activated carbons under the same conditions. The SG-BU material surpasses the activated carbon in the sorption rate of [Au(CN)_2_]^–^ as well as the rate of [Au(CN)_2_]^–^ stripping, which is as little as 15 minutes. This is in striking contrast to the several hours to days needed and used in the case of activated carbon. The [Au(CN)_2_]^–^ stripping was achieved at ambient temperature by aqueous NaCl, while the material was recycled at the same time with 22% efficiency. The [Au(CN)_2_]^–^ stripping from activated carbon is the most expensive step in the gold mining process because it requires high temperatures and pressures, and toxic solutions are also involved.^17^ Moreover, the thermal regeneration of activated carbon is required.^24^ We also demonstrated that SG-BU is selective for Au(CN)_2_]^–^ over [Ag(CN)_2_]^–^, a major contaminant in the gold mining process. Our results demonstrate that a supramolecular-based approach using host-guest binding is an alternative to the conventional processes used in gold mining. Although the SG-BU material offers advantages such as rapid sorption and stripping at ambient temperature and selective uptake of [Au(CN)_2_]^–^ over [Ag(CN)_2_]^–^, activated carbon remains a significantly cheaper alternative with a higher sorption capacity.

This study indicates that the newly developed material, which is based on the BU macrocycle immobilized on silica gel particles, has great potential for the separation of various anions from aqueous solutions, with applications in environmental and analytical chemistry. Experiments on these applications are currently being conducted in our laboratory.

## Data availability

The data supporting this article have been included as part of the ESI.†

## Conflicts of interest

There are no conflicts to declare.

## Supplementary Material

RA-015-D5RA02404H-s001
