# Supplementary material for: Materials utilizing supramolecular host-guest binding for gold extraction
Source: RSC Adv. 2025 Apr 22;15(17):12982–6. doi: 10.1039/d5ra02404h (PMC12013600; doi:10.1039/d5ra02404h)
Supplement: RA-015-D5RA02404H-s001 [file RA-015-D5RA02404H-s001.pdf]

Supplementary Information (SI) for RSC Advances.
This journal is © The Royal Society of Chemistry 2025

Table of Content

1. General Information S2
2. Procedures S3
3. Characterization of SG-BU S4
4. Uptake, Recycling and Striping Experiments S7
5. References S17

1. General Information

Silica gel (Silikagel 60 (0.015-0.040 mm) for column chromatography, product number 1.15111), potassium dicyanoaurate and potassium dicyanoargentate were purchased from Merck and used without further modification. Anion-free dodecabenzylbambus[6]uril (BU) has been synthesized according to a previously published procedure and the spectra were confirmed according to the literature.[1]

The infrared spectra were measured at room temperature in ATR mode using a ZnSe single crystal. The spectra were recorded with an Alpha spectrophotometer (Bruker) and evaluated with OPUS Spectroscopy Software.

The thermogravimetric analyses were carried out in N_2 atmosphere, from room temperature up to 800 °C with a heating rate of 10 °C min^{-1} using Pt crucibles. The measurements were done with Netzsch STA 449C Jupiter (Netzsch).

The TOC analyses were done by Vario TOC cube (Elementar). TOC concentrations were determined from calibration using a standard containing 10 mg/L of TC (5 mg/L of TIC + 5 mg/L of TOC). Samples were prepared in volume of 12 mL, filtered through 0.45 µm PA filters before measurements and injected in a volume of 600 µL.

The UV-Vis measurements were carried out at room temperature using a 2 mm quartz cuvette. The absorption spectra were recorded with a CARY 60 spectrophotometer (Agilent Technologies).

ICP-MS measurements were done by quadrupole ICP-MS Agilent 7900 (Agilent Technologies).

2. Procedures

Silica gel modified with bambusuril, SG-BU

Silica gel (SG) was suspended in chloroform (15 mL) under shaking speed of 180 rpm for 15 min at ambient temperature. **BU** was dissolved in a minimum amount of chloroform (~1.5 mL) and added to the suspension of SG. The suspension was mechanically shaken at room temperature for 30 min at 180 rpm. Then, the solvent was evaporated on a rotary evaporator, and the mixture **SG-BU** was air-dried in a hood at ambient temperature for 24 h.

Sorption kinetics experiment

The contact time needed to reach equilibrium concentration of the anion in solution was examined using UV-Vis spectroscopy. The **SG-BU** (9:1, 100 mg) material was placed in aqueous potassium dicyanoaurate solution (1 mM, 3 mL) and shaken (250 rpm) for a certain time. After a given time, the SG-BU was left to sediment, and an absorption spectrum of the supernatant was measured on a UV-Vis spectrophotometer. The actual concentration of the anion in solution was subsequently calculated from a calibration curve.

Langmuir isotherm of sorption

The maximum amount of dicyanoaurate sorbed by **SG-BU** was examined using UV-Vis spectroscopy, when different concentrations of aqueous potassium dicyanoaurate solution (3 mL) were used (varied from 0 – 2 mM). The samples **SG-BU** (9:1) (10 mg) were shaken for 30 min at ambient temperature. The adsorption data of Au on **SG-BU** were fitted with Langmuir equation. The application of Langmuir model is widely used for the purpose of sorption parameters determination.[2–4]

Anion uptake experiments

The ability of the system to capture anions from the aqueous solution was examined using UV-Vis spectroscopy and ICP, respectively. Certain amount of **SG-BU** was placed in a vial and potassium dicyanoaurate aqueous solution (1 mM, 3 mL) was added. The system was shaken for 15 min at 250 rpm at ambient temperature. Subsequently, **SG-BU** was left to sediment and afterwards, the supernatant was analysed.

Recycling experiments

SG-BU after anion uptake was washed twice with 9 mL of NaCl solution (3.5 wt.%) for 15 min at 250 rpm. The recycled substrate was then treated again with anion solution (1 mM, 3 mL, 15 min).

All the experiments were repeated at least twice, and given data are presented as mean values ± standard deviations.

3. Characterization of SG-BU

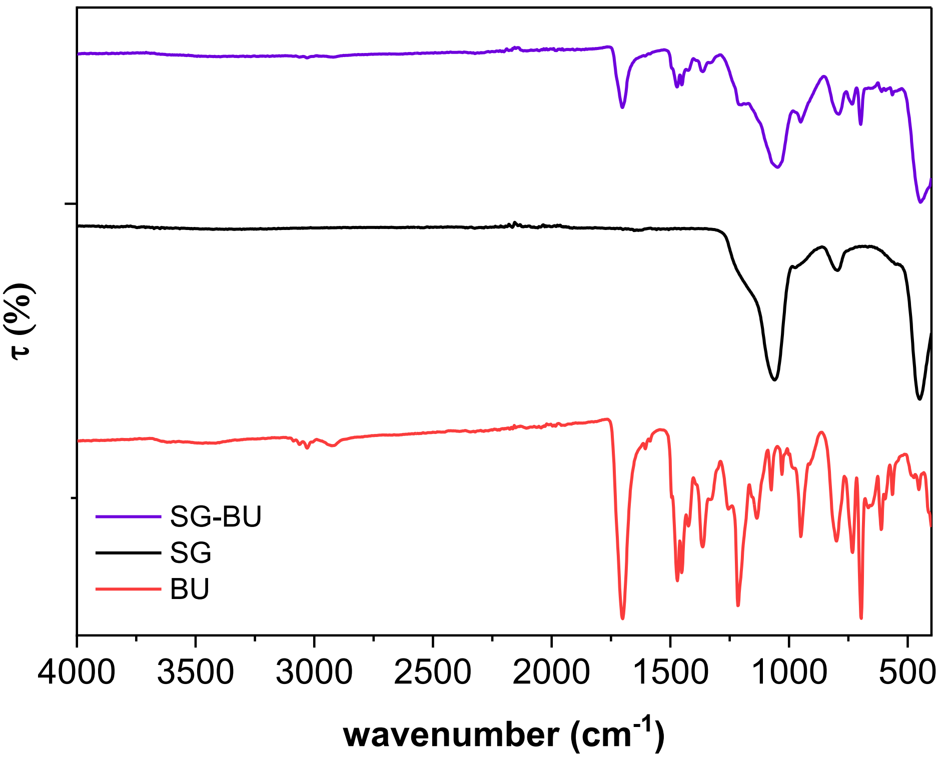

Figure S1. ATR spectra of SG, BU and **SG-BU** (9:1).

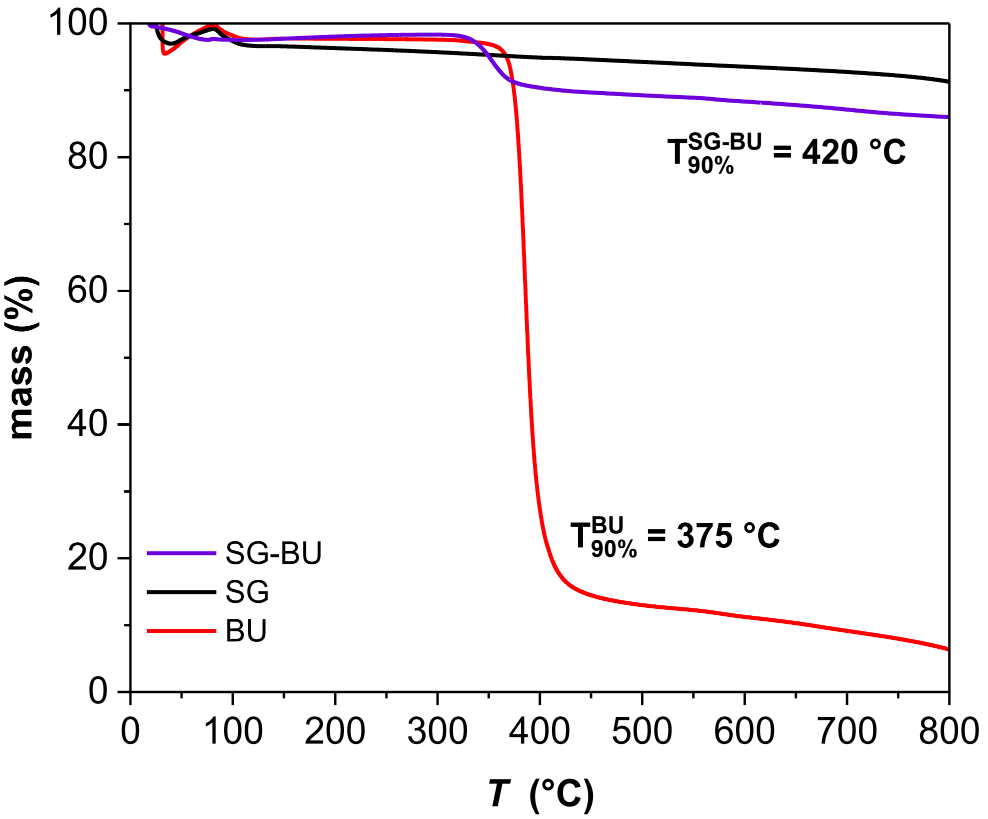

Figure S2. Thermogravimetric measurements for SG, BU and **SG-BU** (9:1).

Table S1. TOC analyses of materials.

	Dispersed material (g/L)	TOC concentration (mg/L)
water	-	2.5 ± 0.11
BU	4.09 ± 0.4	4.3 ± 0.05
SG	30.38 ± 0.8	3.7 ± 0.25
SG-BU (9:1)	31.42 ± 0.4	3.4 ± 0.77
$K[Au(CN)_2]$ (1 mM)	–	18.9 ± 0.20
SG-BU (9:1) + $[Au(CN)_2]^-$	20.20 ± 0.1	17.1 ± 0.45
NaCl (10 mM)	–	2.2 ± 0.19
SG-BU (9:1) + Cl^-	20.75 ± 0.1	3.85 ± 0.05

The TOC analyses were carried out to confirm the insolubility of **BU** in water alone and in the form of SG-BU (9:1) material. Materials were placed in milliQ water in the absence and the presence of $K[Au(CN)_2]$ or NaCl. The mixtures were shaken for 120 min and water content was then analyzed. The analyses revealed that the similar low TOC values compared to that of blank water sample. Higher TOC values in the presence of $K[Au(CN)_2]$ correspond to the presence of carbon in the anion. The obtained results show that **BU** is insoluble in water, and it remains attached to the surface of SG in aqueous solutions under different conditions.

4. Uptake, Recycling and Striping Experiments

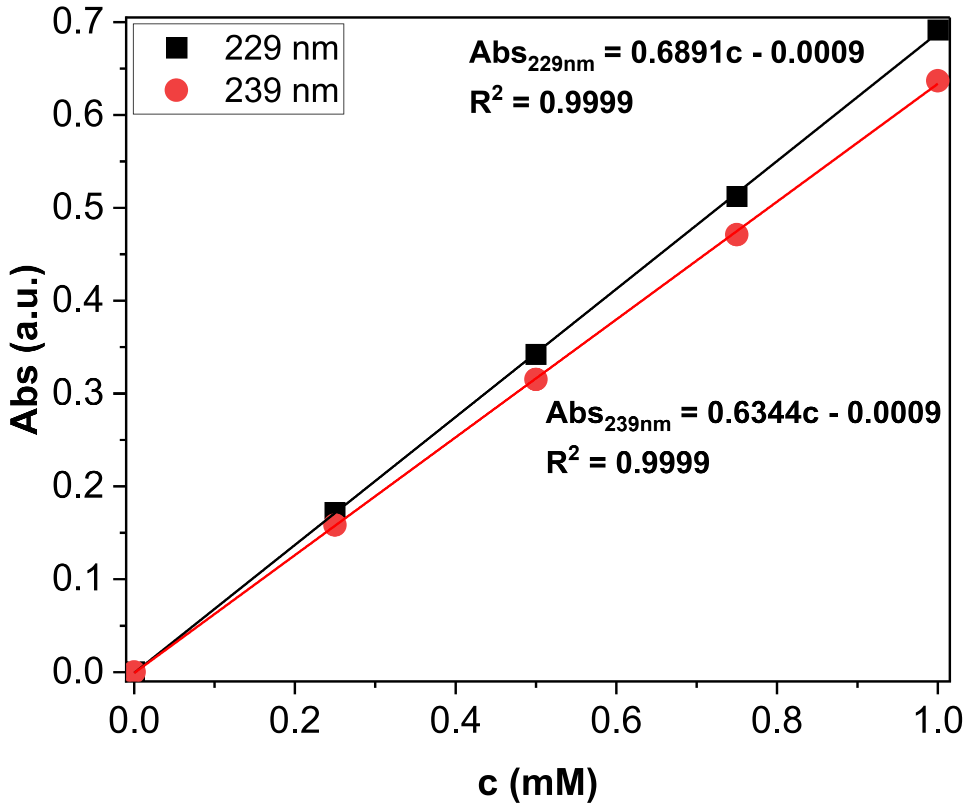

Figure S3. Calibration curves of dicyanoaurate used for calculation of its actual concentration in solution.

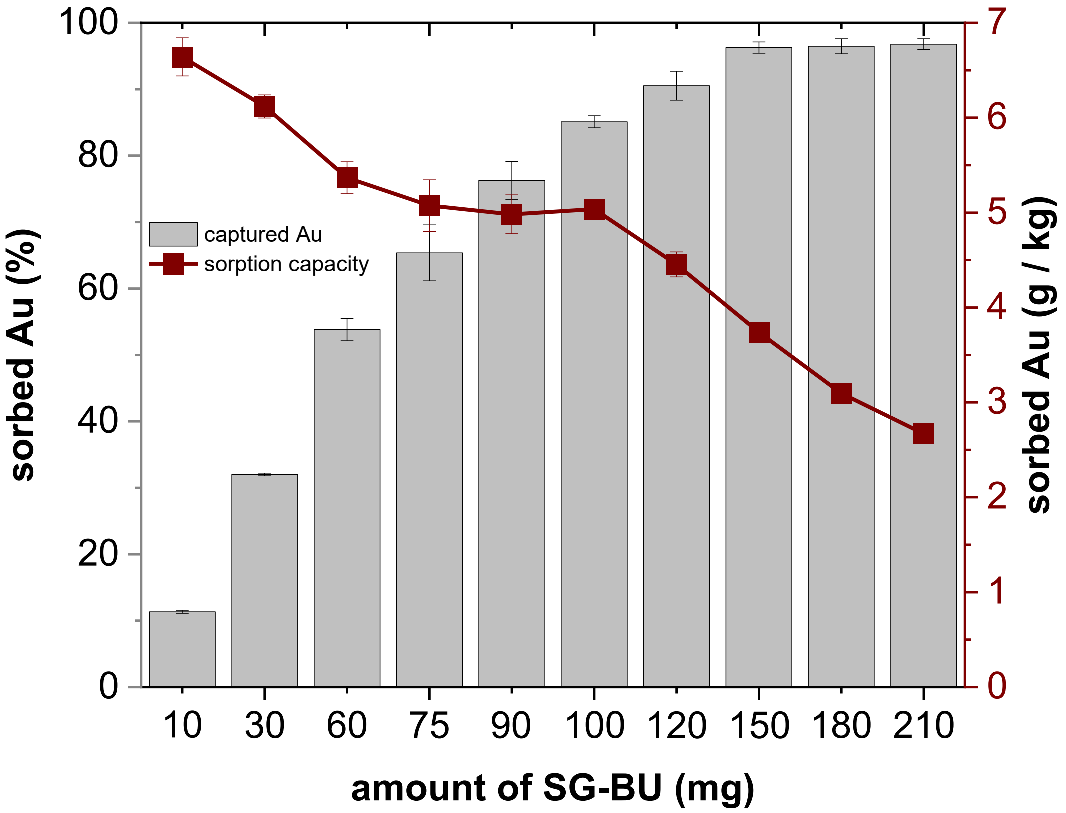

Figure S4. Influence of the amount of **SG-BU** (9:1) on anion uptake and sorption capacity of material.

Increasing amount of SG-BU (9:1) material was added to water solution of $K[Au(CN)_2]$ (1.0 mM, 3.0 mL, 3.0 µmol) and amount of sorbed anion was calculated from UV-vis spectra.

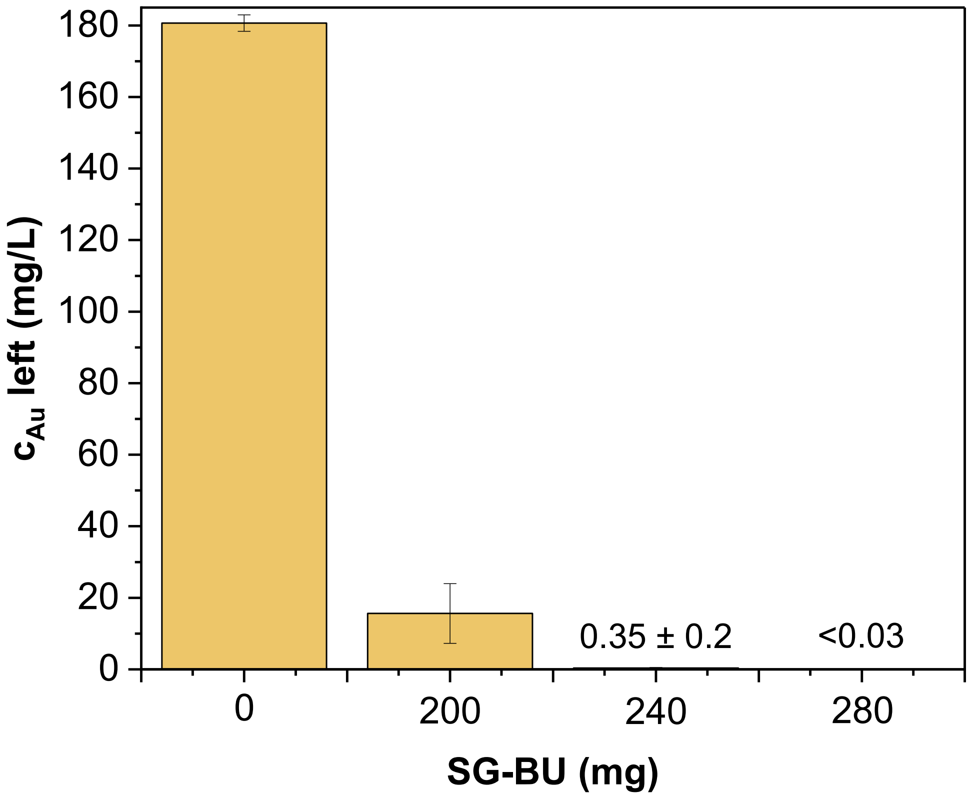

Figure S5. Influence of the amount of **SG-BU** (9:1) on anion uptake. Data obtained from ICP.

Increasing amount of SG-BU (9:1) material was added to water solution of $K[Au(CN)_2]$ (1.0 mM, 3.0 mL, 3.0 µmol) and amount of anion remaining in the solution was monitored by inductively coupled plasma (ICP) spectroscopy.

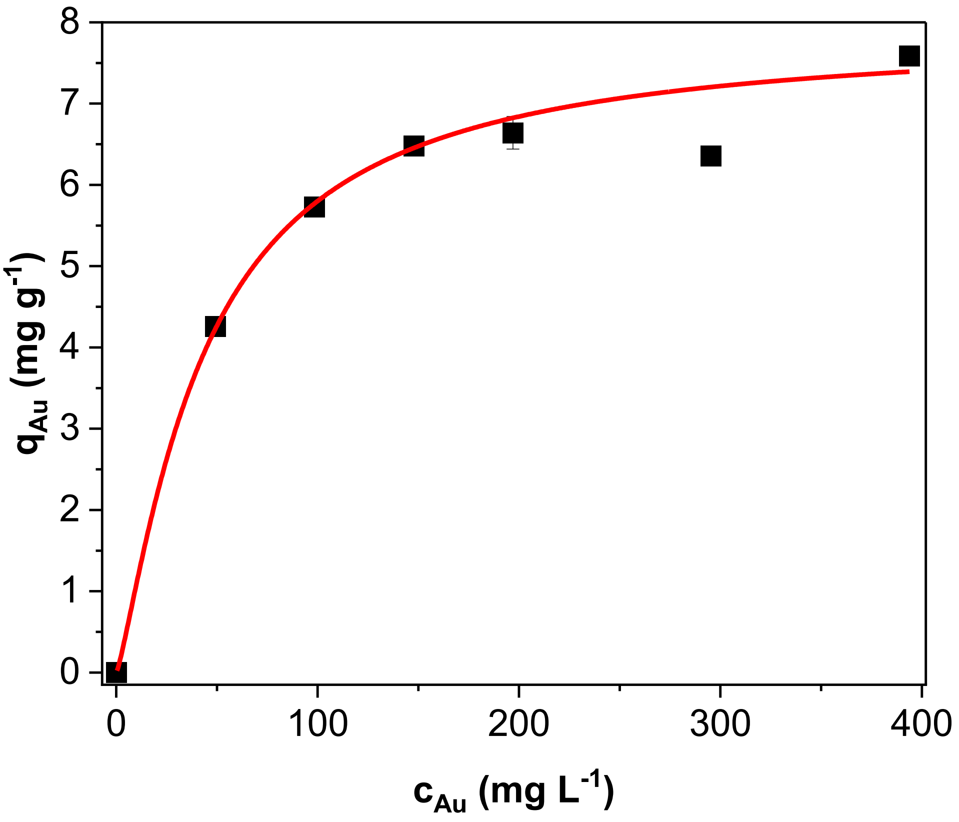

Figure S6. Langmuir-type isotherm for the sorption of $K[Au(CN)_2]$ from its water solutions by **SG-BU** (9:1) material.

The Langmuir isotherm provided sorption parameters, such as maximum sorption capacity q_m of SG-BU (9:1) material, and association constant K_L according to following equation:

$$q_{Au} = c_{Au} \frac{q_m K_L}{1 + K_L c_{Au}}$$

The maximum sorption capacity value for Au was q_m = 7.87 ± 0.70 mg g^{-1} (= 39.98 ± 3.5 µmol g^{-1}).

The association constant value for Au resulted in K_L = 0.03 ± 0.002 L mg^{-1}, which gives logK_L = 3.77 M^{-1}.

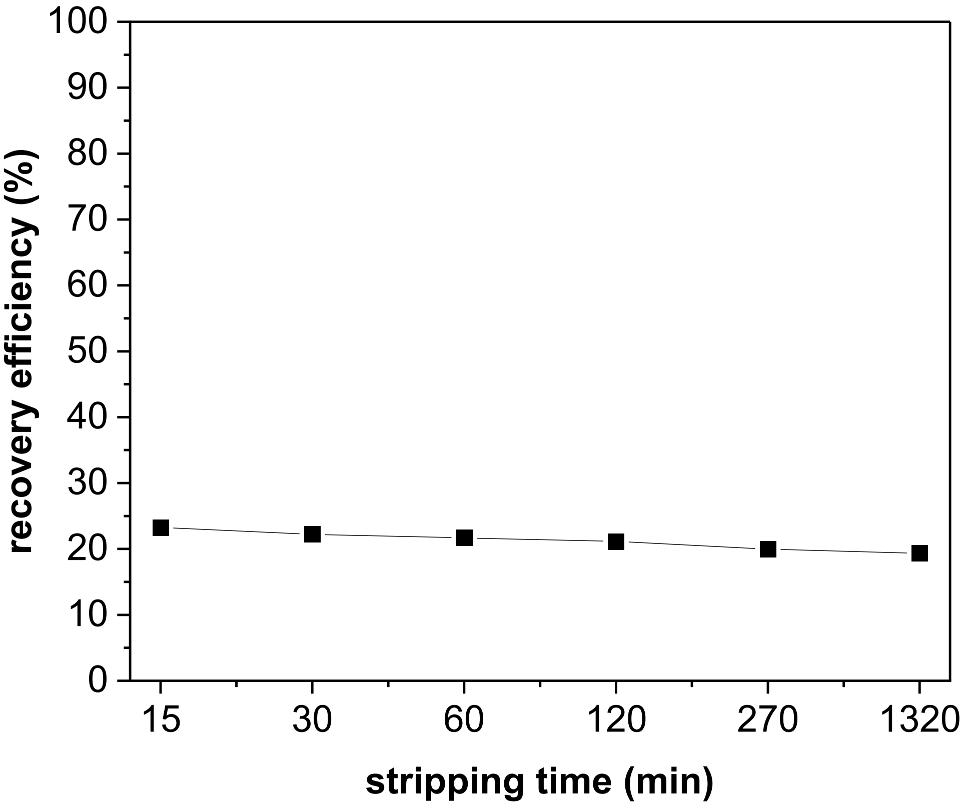

Figure S7. Monitoring of recovery efficiency of dicyanoaurate from **SG-BU** (9:1, 100 mg) over time.

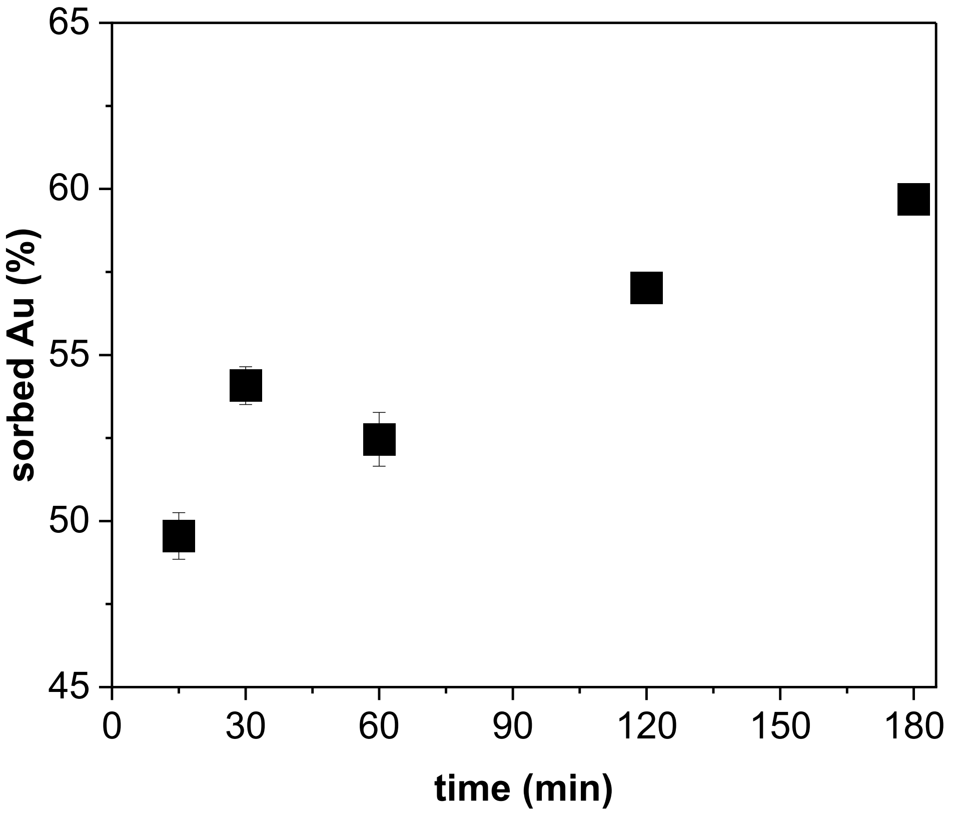

Figure S8. Sorption kinetics expressed as time dependence of the sorption efficiency (the weight fraction of gold removed from the solution (1 mM, 3 ml) by activated carbon (10 mg).

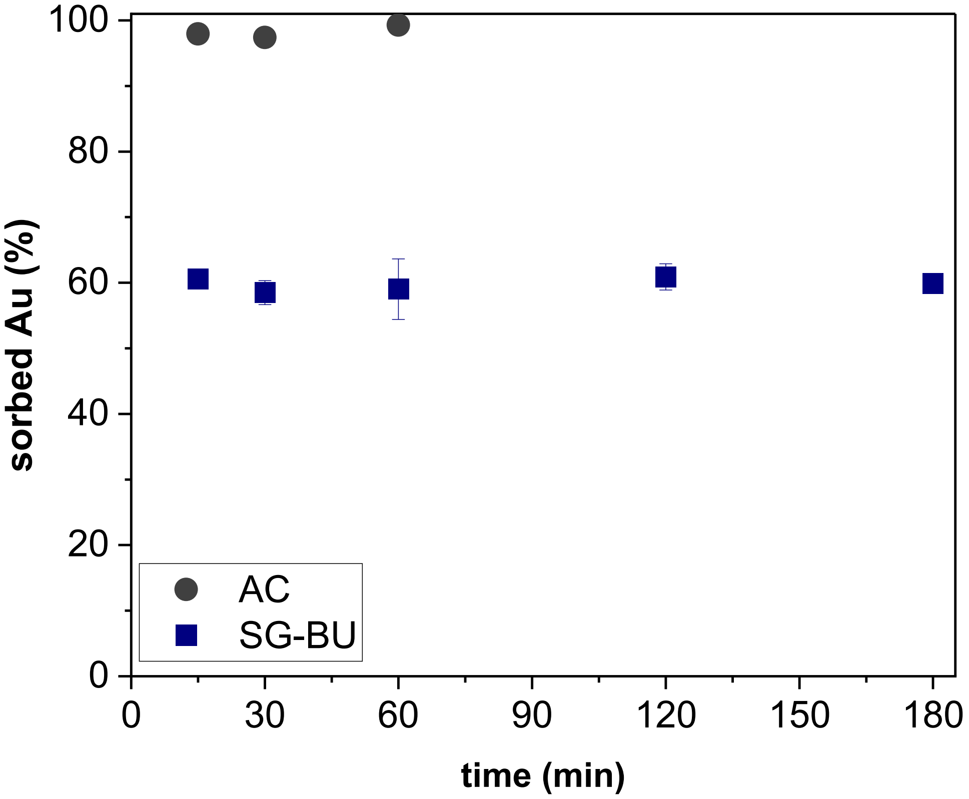

Figure S9. Sorption kinetics expressed as time dependence of the sorption efficiency (the weight fraction of gold removed from the solution (0.1 mM, 3 ml) by the SG-BU (9:1) and activated carbon (10 mg).

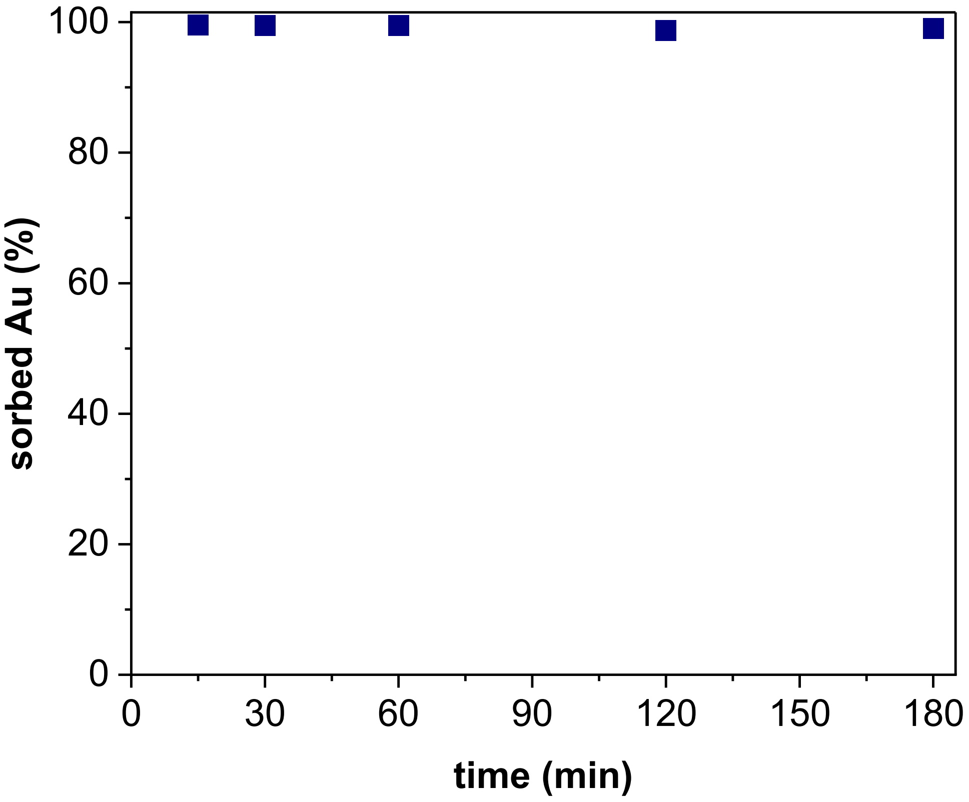

Figure S10. Sorption kinetics expressed as time dependence of the sorption efficiency (the weight fraction of gold removed from the solution (0.1mM, 3 ml) by the **SG-BU** (9:1) (100 mg).

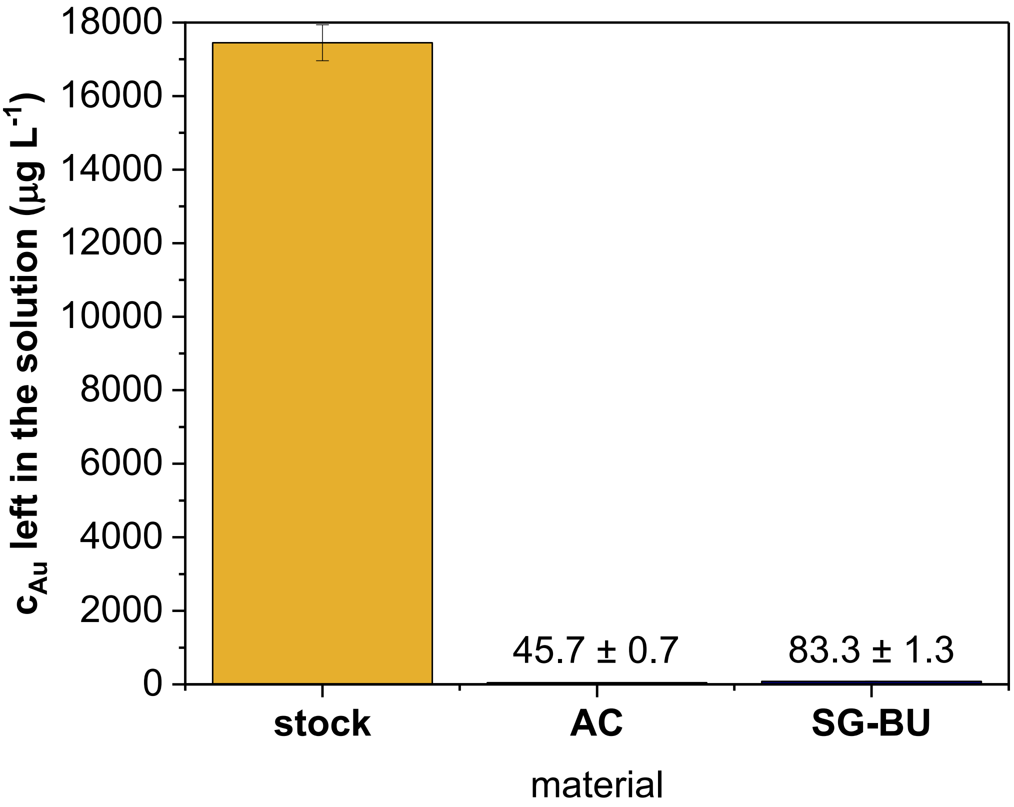

Figure S11. Anion uptake from 0.1 mM solution (stock) by activated carbon (AC, 10 mg) and **SG-BU** (9:1) (100 mg). Data obtained from ICP.

Quantitative uptake of gold from 0.1 mM solution was observed by ICP. In the case of activated carbon, 10 mg removed 99.7 %, while in the case of **SG-BU** (9:1) 100 mg removed 99.5 % of anion.

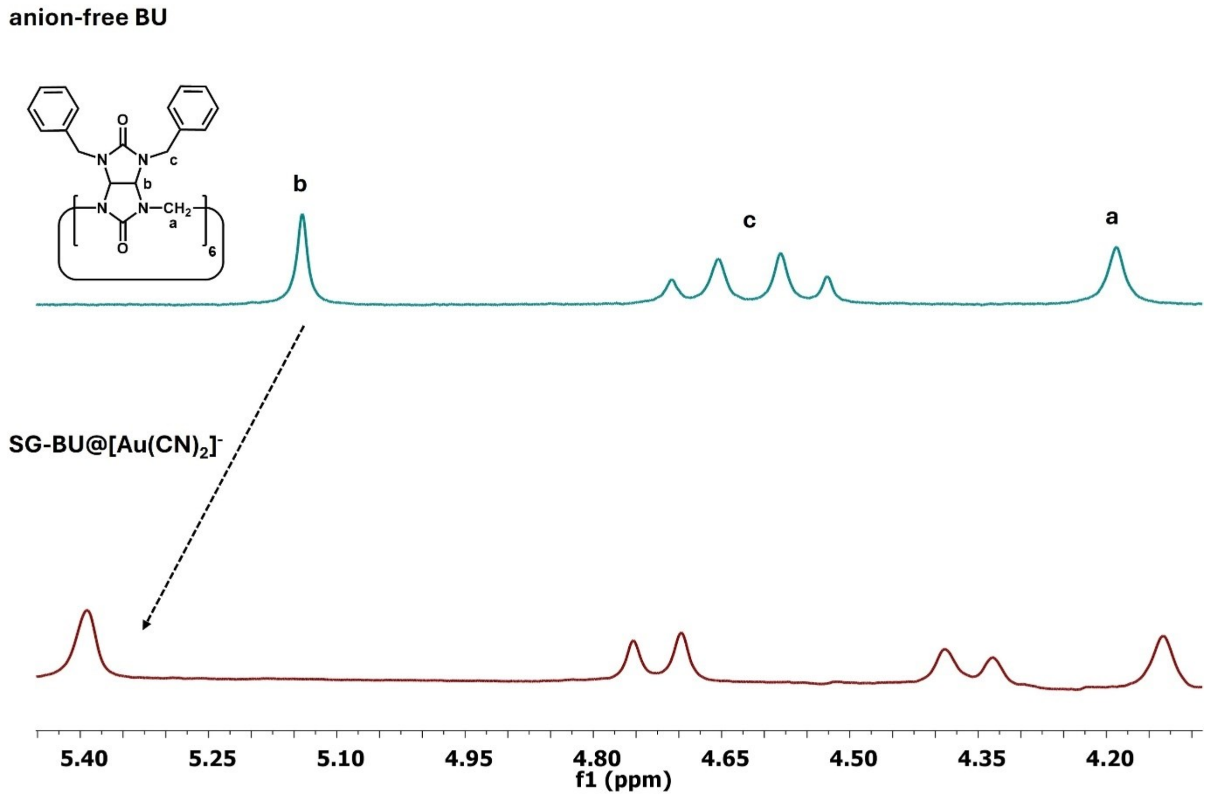

Figure S12. ^{1}H NMR spectra (300 MHz, DMSO-D$_6$, 25 °C) of anion-free BU (top) and BU obtained from the SG-BU (9:1) material (bottom).

We conducted an experiment in which the SG-BU material was treated with an aqueous solution of $[Au(CN)_2]^-$. The material was then collected by filtration and dried. Upon treatment with DMSO-D$_6$, the bambusuril-$[Au(CN)_2]^-$ complex attached to the material got dissolved in the solvent, and the solution was analyzed by ^{1}H NMR spectroscopy. The observed chemical shifts of bambusuril signals were consistent with the typical pattern known for its anion complexes, particularly marked by a significant downfield shift of the methine protons (b) compared to those of an anion-free BU. This confirms the formation of a host-guest complex between BU and $[Au(CN)_2]^-$ during the sorption.

5. References

1 V. Havel, J. Svec, M. Wimmerova, M. Dusek, M. Pojarova and V. Sindelar, Bambus[n]urils: a New Family of Macrocyclic Anion Receptors, *Org. Lett.*, 2011, **13**, 4000–4003.

2 K. K. H. Choy, J. F. Porter and G. McKay, Langmuir Isotherm Models Applied to the Multicomponent Sorption of Acid Dyes from Effluent onto Activated Carbon, *J. Chem. Eng. Data*, 2000, **45**, 575–584.

3 J. Xia, H. Mahandra and A. Ghahreman, Efficient Gold Recovery from Cyanide Solution Using Magnetic Activated Carbon, *ACS Appl. Mater. Interfaces*, 2021, **13**, 47642–47649.

4 L. Yang, F. Jia, B. Yang and S. Song, Efficient adsorption of Au(CN)2− from gold cyanidation with graphene oxide-polyethylenimine hydrogel as adsorbent, *Results Phys.*, 2017, **7**, 4089–4095.
